# Radical Dendrimers Based on Biocompatible Oligoethylene Glycol Dendrimers as Contrast Agents for MRI

**DOI:** 10.3390/pharmaceutics12080772

**Published:** 2020-08-14

**Authors:** Songbai Zhang, Vega Lloveras, Daniel Pulido, Flonja Liko, Luiz F. Pinto, Fernando Albericio, Miriam Royo, José Vidal-Gancedo

**Affiliations:** 1 Institut de Ciència de Materials de Barcelona (ICMAB-CSIC) and CIBER-BBN, Campus Universitari de Bellaterra, Bellaterra, 08193 Barcelona, Spain; szhang@icmab.es (S.Z.); vega@icmab.es (V.L.); flonjaliko@yahoo.com (F.L.); luizpinto@sapo.pt (L.F.P.); 2 Institut de Química Avançada de Catalunya (IQAC-CSIC) and CIBER-BBN c/ Jordi Girona 18–26, 08034 Barcelona, Spain; daniel.pulido@iqac.csic.es (D.P.); albericio@ub.edu (F.A.); miriam.royo@iqac.csic.es (M.R.); 3 Department of Inorganic and Organic Chemistry, University of Barcelona, 08028 Barcelona, Spain; 4 School of Chemistry and Physics, University of KwaZulu-Natal, Durban 4041, South Africa

**Keywords:** contrast agent, magnetic resonance imaging (MRI), organic radicals, PROXYL, OEG dendrimers, radical dendrimers, relaxivity, Gd-DTPA, infectious diseases

## Abstract

Finding alternatives to gadolinium (Gd)-based contrast agents (CA) with the same or even better paramagnetic properties is crucial to overcome their established toxicity. Herein we describe the synthesis and characterization of entirely organic metal-free paramagnetic macromolecules based on biocompatible oligoethylene glycol dendrimers fully functionalized with 5 and 20 organic radicals (OEG Gn-PROXYL (*n* = 0, 1) radical dendrimers) with the aim to be used as magnetic resonance imaging (MRI) contrast agents. Conferring high water solubility on such systems is often a concern, especially in large generation dendrimers. Our approach to overcome such an issue in this study is by synthesizing dendrimers with highly water-soluble branches themselves. In this work, we show that the highly water-soluble OEG Gn-PROXYL (*n* = 0, 1) radical dendrimers obtained showed properties that convert them in good candidates to be studied as contrast agents for MRI applications like diagnosis and follow-up of infectious diseases, among others. Importantly, with the first generation radical dendrimer, a similar *r*_1_ relaxivity value (3.4 mM^−1^s^−1^) in comparison to gadolinium-diethylenetriamine pentaacetic acid (Gd-DTPA) used in clinics (3.2 mM^−1^s^−1^, r.t. 7T) has been obtained, and it has been shown to not be cytotoxic, avoiding the toxicity risks associated with the unwanted accumulation of Gd in the body.

## 1. Introduction

Infectious diseases are the second leading cause of death in the world. Noninvasive imaging research allows deepened information through longitudinal studies of animal models of human diseases. Such studies have greatly contributed to the understanding of pathogenesis and preclinical investigations on drug development [1].

To increase diagnostic and therapeutic treatments it would be very important to have a diagnostic imaging tool that detects pathogens non-invasively and differentiates between bacterial infections and other sources of inflammation. Some imaging techniques have been developed and applied to different models of infection like positron emission tomography (PET), bioluminescence (BLI) and fluorescence imaging, single photon emission computed tomography (SPECT), X-ray based computed tomography (CT) and magnetic resonance imaging (MRI) [2,3,4,5,6]. Among them, MRI is the most versatile and widely used clinical diagnostic tool nowadays, showing some advantages over the others. It provides images of soft tissue anatomy in excellent detail, with high spatial resolution (∼100 µm), unlimited penetration depth, long effective imaging window, rapid in vivo images acquisition and absence of ionizing radiation. This method is largely used for non-invasive diagnostic routine detection and staging of cancer, pathologies of the brain such as stroke or neurodegenerative disease, in cardiac imaging and others. For the diagnosis of infectious disease, either viral or bacterial, MRI is used to detect local inflammation and other manifestations of the immune response thanks to changes in tissue parameters such as relaxation time, diffusivity or water content [2,3,7]. Tissue differences in such parameters can be detected by T_1_, T_2_, proton density or gradient echo MR sequences. However, the sensitivity of MRI is generally lower as compared to SPECT and bioluminescence. For this reason, the development of MRI contrast agents (CA) with high efficiency and sensitivity is essential in imaging. To induce significant tissue contrast at the site of infection, contrast agents are increasingly used in studies of infectious disease. The principal contrast agents used currently are based on iron oxide particles and lanthanide chelates (basically gadolinium-based contrast agents). The development of such contrast agents as well as fluorinated compounds has allowed researchers to specifically label immune cells and receptors at the site of infection [8,9,10,11,12]. In this way, the study of host–response mechanisms and the development of inflammation becomes possible.

Iron-oxide-based particles are contrast agents that shorten transverse relaxation time of water protons, leading to negative contrast in T2* images. Often, macrophages are labeled with superparamagnetic iron oxide (SPIO) particles to study them at the site of infection [13,14] and some works have reported a method to label and track bacteria directly by MRI. However, an important disadvantage of the T2* detection of SPIOs is that sometimes the negative contrast is difficult to distinguish from signal voids due to susceptibility artifacts or field inhomogeneities, among others [15].

On the other hand, gadolinium-based contrast agents are widely used as positive contrast agents in MRI, shortening T1 relaxation time and leading to high signal enhancement because their seven unpaired electrons (spin 7/2) provide Gd^3+^ with an important magnetic moment [16,17]. A paramagnetic relaxation enhancement is achieved due to dipole–dipole interactions with the surrounding water protons. Almost 40 years ago, Gd-compounds were used to detect zones of abdominal inflammation. More recently, Gd-complexes have been widely used in the evaluation of cerebral infection and loss of the blood–brain barrier, becoming a well-established technique. Gd-complexes are able to pass the vessel wall only in zones of inflammation, leading to increased T1 relaxation in the contiguous tissue [18,19,20]. Usually, Gd-complexes are bioconjugated to different kind of antibodies to target inflammatory or immunological processes [21]. Furthermore, Gd-based dendrimers have been developed as biomarkers [22]. Very recently, in the COVID-19 pandemic, although it is not the most common tool applied for COVID-19 diagnosis, MRI has been used to study the vulnerability of different organs for a better understanding of the pathogenesis and mechanisms of SARS-CoV-2 infection. Such infection, although mainly distributed in the lung, has been shown through minimally invasive autopsies to also cause damage in the heart, vessels, liver, kidney, and other organs [23].

Although gadolinium-based contrast agents have historically been considered as safe, it is recognized to be involved with the relationship between the development of nephrogenic systemic fibrosis in patients with severe renal impairment following administration. In addition to this, the accumulation of toxic Gd(III) ions in the brain, liver, bones, skin or kidneys of patients with normal renal function has recently been demonstrated. For this reason, it is very important to find alternative imaging probes with the same or even better paramagnetic properties than current Gd-based CA.

Our group has developed entirely organic metal-free contrast agents based on dendrimers fully functionalized with organic radicals (radical dendrimers) to this end [24]. In fact, stable organic free radicals, such as nitroxides, are paramagnetic species that were investigated as T_1_ CAs for MRI many years ago [25,26,27]. However, their inherent low paramagnetic relaxivity (spin ½) and rapid bioreduction avoided their widespread application. With the incorporation of many nitroxyl units to a dendrimer, a higher molecular relaxivity can be achieved and, additionally, a protective shield effect can be provided to the radicals by restricting the fast access of reducing agents. Dendrimers are a very special class of hyperbranched macromolecules with globular structures characterized by the strict control over their structure, making them nearly perfect monodisperse macromolecules. Dendrimers can be generated with a wide range of scaffold structures, sizes and surface functionalities making them excellent candidates to hold paramagnetic organic radicals, leading to a variety of radical dendrimers with different sizes. The control over such parameters opens the opportunity to modulate their biodistribution and pharmacokinetics.

Only a few reports based on dendrimers fully functionalized with organic radicals have been reported previously [28,29,30,31,32,33,34,35,36,37,38,39,40], with most of them devoted to the study of their magnetic and electronic properties and only few of them describing MRI CA applications [30,31,32]. In those very few reports, PAMAM and PPI dendrimers were functionalized with 2,2,5,5-tetramethylpyrrolidin-1-oxyl (PROXYL) radical units, or nitronyl nitroxides radicals, resulting in high paramagnetic systems. However, the authors pointed out aggregation problems in the water phase of such nitroxide-based dendrimers for their limited water solubility. In fact, because the hydrophobicity of the surface of the nitroxide-covered dendrimer may contribute to their limited applications in MRI, some approaches to increase water solubility had to be found. Water solubility is an essential property for their in vivo applications but it is usually an issue difficult to solve, especially in large generation dendrimers. One strategy used to overcome such a problem was including water-solubilizing groups such as poly (ethylene glycol) (PEG) chains on the surface of the dendrimer, like Rajca [41] and Katayama [42] and co-workers did, respectively. However, in that way, the total number of nitroxides anchored on the surface decreases as not all the branches are free to be spin labeled and hence the molecular relaxivity should be lower than with a fully nitroxide covered surface. In addition, control over the number and density of the anchored nitroxides is more difficult. We recently proposed another strategy to increase water solubility and, at the same time, maintain the entire number of terminal functional groups to be functionalized [24]. It was an innovative procedure consisting of the use of an amino acid as linker between the dendrimer branches and the organic radicals, which provided an available amino group for radical coupling, and a methyl ester group to afford negative charges via hydrolysis, conferring high solubility at physiological pH. In that way, we obtained a series of radical dendrimers fully soluble in water with up to 48 PROXYL radicals that provided very high relaxivities.

**Scheme 1:**
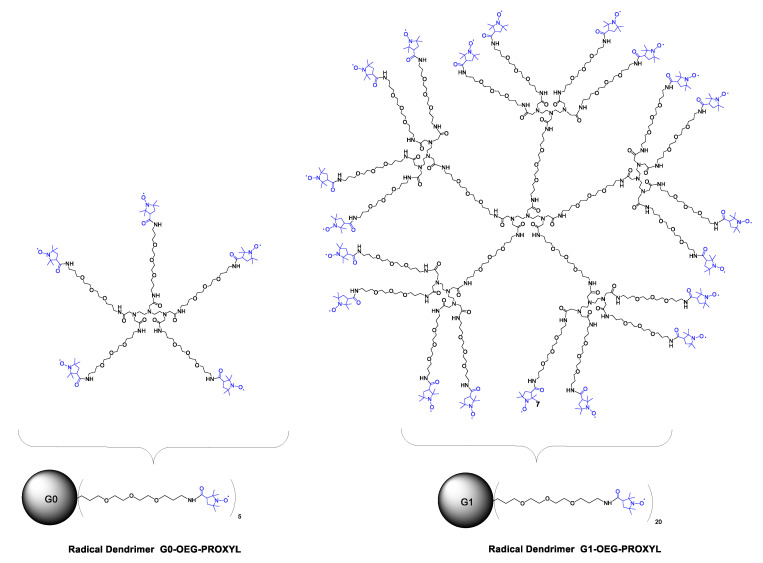
Structures of Gn-OEG-PROXYL radical dendrimers, *n* = 0, 1.

In the present work, we propose another way to obtain fully water-soluble radical dendrimers, also allowing at the same time the full functionalization of their branches. It consists of the use of dendrimers containing water-soluble branches themselves [43,44]. We have synthesized two generations of DTPA (diethylenetriaminepentaacetic acid)-core based dendrimers containing five [45] and twenty equivalent oligoethylene glycol (OEG) branches. The amino functional groups at the end of each branch (4,7,10-trioxa-13-tridecanamine) have permitted us to fully functionalize both generations with PROXYL radical units (Gn-OEG-PROXYL dendrimers, *n* = 0, 1; Scheme 1). The obtained radical dendrimers showed high molecular relaxivity and full solubility at physiological pH. The control over the size of the dendrimers opens the opportunity to modulate their distribution profile in the body, which is impossible in the case of Gd-DTPA. In addition, it is important to highlight that such OEG dendrimers used as scaffolds are biocompatible [46]. The fact that OEG dendrimers are biocompatible and that their small size minimizes their unwanted accumulation in the body gives the possibility to obtain contrast agents which improve the properties of Gd-based CA. It is worth saying that to the best of our knowledge, this is the first time that OEG dendrimers are functionalized with radical units and designed for MRI applications. Their magnetic properties were investigated by EPR, followed by the evaluation of their relaxivity and cytotoxicity, demonstrating properties that designate them as potential compounds to be studied as an alternative to Gd-based CA for MRI applications such as diagnosis and follow-up of infectious diseases, among others.

## 2. Materials and Methods

### 2.1. Materials

All reactants, unless stated otherwise, were purchased from Sigma Aldrich Inc. (St. Louis, MO, USA) at the highest purity available and used without further purification. 1-(*tert*-butyloxycarbonyl-amino)-4,7,10-trioxa-13-tridecanamine and PyBOP were purchased from Iris Biotech (Marktredwitz, Germany). DMF, CH_2_Cl_2_, methanol (HPLC grade), dioxane, AcOEt, were obtained from SDS-Carlo Erba (Sabadell, Spain). CH_2_Cl_2_ was distilled from CaH_2._

### 2.2. Methods

**Chromatography**. Thin layer chromatography (TLC) was performed on Merck 60F254 silica plates or on Fluka aluminium oxide plates visualized by UV (254 nm) or by ninhydrin. Analytical RP-HPLC was carried out on a XBridgeTM C_18_ reversed-phase analytical column (3.5 μm × 4.6 mm × 100 mm).

**HPLC-MS** analyses were carried out on a Waters instrument (Milford, MA, USA) comprising a separation module (Waters 2795), an automatic injector, a photodiode array detector (Waters 2996), and a Waters ZQ 4000 mass detector. HPLC columns were obtained from Waters. Data were managed with MassLynx V4.1 software (Waters). High-resolution mass spectroscopy (HRMS) results were obtained using an LC/MSD-TOF spectrometer from Agilent Technologies Inc. (Santa Clara, CA, USA).

**Nuclear magnetic resonance spectroscopy (NMR)**^1^H NMR (400 MHz) spectroscopy was performed on a Varian Mercury 400 MHz instrument. Chemical shifts (δ) are expressed in parts per million downfield from tetramethylsilyl chloride. Coupling constants are expressed in Hertz. The following abbreviations are used to indicate multiplicity: s: singlet; d: doublet, t: triplet, q: quartet, qt: quintet, m: multiplet, and bs: broad signal. ^1^H NMR (250 MHz) of G0-OEG-PROXYL with acid ascorbic excess was performed at Bruker (Rheinstetten, Germany) spectrometer Avance DRX-250.

**Mass spectrometry**. For compounds 1 to 7, mass spectra were performed on an Applied Biosystems VoyagerDE RP instrument, using α-cyano-4-hydroxycinnamic acid matrix (Sigma Aldrich, St. Louis, MO, USA). For radical dendrimers, a matrix-assisted laser desorption/ionization-time-of-flight mass spectrometer (MALDI-TOF) Bruker Daltonic system was used. Dithranol was used as matrix. In an Eppendorf tube, 4 µL of the radical dendrimer solution in dichloromethane was mixed with 4 µL of the matrix (Dithranol, Sigma Aldrich, St. Louis, MO, USA) dissolved in dichloromethane. An aliquot of the mixture was placed in a well of the plate and left to evaporate. Positive mode was used in the analysis.

**Ultrafiltration** of radical dendrimers was performed on solvent-resistant stirred cells from EMD Millipore (Billerica, MA, USA) with regenerated cellulose membranes. Ultrapure water (Milli-Q, EMD Millipore) or mixtures of ultrapure water and HPLC grade acetone were used for ultrafiltration.

**Size exclusion chromatography (SEC)** analysis was carried out using an Agilent 1260 infinity II liquid chromatography system apparatus equipped with a diode array detector under the following conditions: a PSS Suprema pre-column (10 μm, 8 × 50 mm) and a PSS Suprema analytical column (10 μm, 100 Å, 8 × 300 mm) with a diode array detector were used. LiCl in water (0.25 mM) was used as eluent at a flow rate of 0.5 mL/min at 35 °C. Radical dendrimers were dissolved in the eluent to reach a final concentration of 1 mg/mL and filtered through 0.2 μm nylon filter before injection.

**Electron paramagnetic resonance spectroscopy (EPR)** spectra were obtained with an X-Band (9.4 GHz) Bruker ELEXSYS E-500 spectrometer equipped with a ST8911 microwave cavity, a Bruker variable temperature unit, a field frequency lock system Bruker ER 033 M and with a NMR Gaussmeter Bruker ER 035 M. The modulation amplitude was kept well below the line width, and the microwave power was well below saturation. Microwave power: 0.62 mW, modulation amplitude: 0.6 G, line width: 1.2 G (alternate lines: 1.8 G). All samples were previously degassed with Ar. EPR spectra in aqueous phase were carried out with a flat quartz EPR cell.

**FT-IR** spectra were recorded in a FT/IR-4700 from JASCO (Tokyo, Japan) with an ATR (attenuated total reflectance) accessory, in the 400–4000 cm^−1^ range with 4 cm^−1^ resolution.

**Dynamic light scattering (DLS)** experiments were carried out on a Nano-S Zetasizer (Malvern Instrument Ltd., Malvern, UK) with back scattering detector (173°, 633 nm laser wavelength) to measure hydrodynamic size (diameter) in batch mode at 25 °C. Dendrimer samples were prepared at a concentration of 1 mg/mL in 30 mM phosphate buffer pH 7.4. The samples were filtered through 0.2 μm PTFE filter before analysis. A minimum of 3 measurements per sample were made. Hydrodynamic size was reported as the volume-weighted average.

**Magnetic resonance imaging (MRI)** experiments were carried out in a BioSpec 70/30 Bruker system using a 7.0 T horizontal-bore superconducting magnet equipped with actively shielded gradients (B-GA12 gradient coil inserted into a B-GA20S gradient system). A quadrature 72 mm inner diameter volume coil was used for in vitro studies.

**Relaxometric measurements**. Longitudinal (*r*_1_) and transverse (*r*_2_) relaxivities were determined per concentration of PROXYL units. Different concentrations of PROXYL radicals in 30 mM phosphate buffer (pH 7.4) were prepared. Relaxivity measurements were obtained at room temperature. The software used for the calculations of T_1_ and T_2_ relaxations was Paravision 6.0 (Bruker Software). *r*_1_ *relaxivity*. Series of axial T_1_-weighted (T1W) images were acquired for each concentration of PROXYL to obtain T_1_ maps based on a magnetization saturation experiment and the following parameters: repetition time (TR) = 70−6000 ms, echo time (TE) = 9.2 ms, field of view (FOV) = 2.5 × 2.5 cm, averages (Av) = 1, acquisition matrix (Mtx) = 128 × 128. The T_1_ values were calculated from the mean signal in the region of interest (ROI) for each repetition time, adjusted to the equation: *S* = *So* [1 − (−TR/T1)]. *r*_2_ *relaxivity*. T_2_ maps were calculated from multi spin echo images with a TR of 2 s. A total of 25 echo images were acquired with a TE of 8.02 ms, which was also the interval time between echo image acquisitions. T_2_-map parameters were as follows: 1 axial slice of 2 mm thickness; TR = 3200 ms; TE = 8.02 ms (25 echo times with interval of 8.02 ms in between), 1 average. Field of view (FOV): 35 × 35 mm; Mtx: 256 × 256. The T2 values were calculated from mean signal in the ROI for each echo image, adjusted according to the equation: *S* = *So* (−TE/T_2_)].

**In vitro cytotoxicity** assays were conducted with a Vero cell line (ATCC^®^ CCL-81™). Vero cell line was initiated from the kidney of a normal adult African green monkey. G0- and G1-OEG-PROXYL samples were filtered and serially diluted in DMEM+10%PBS media. Then, the cells were mixed in each solution (1 × 10^4^ cells/well) to avoid pipetting errors. The dilutions, control and blank were seeded. The XTT kit (CyQUANT™ XTT Cell Viability Assay) was added at 24 h and the reading was performed 4h after the tetrazolium was added. From each concentration quadruplicates were prepared and measured.

## 3. Results and Discussion

### 3.1. Synthesis of G0- and G1-OEG-NH_2_ Dendrimers

**Scheme 2:**
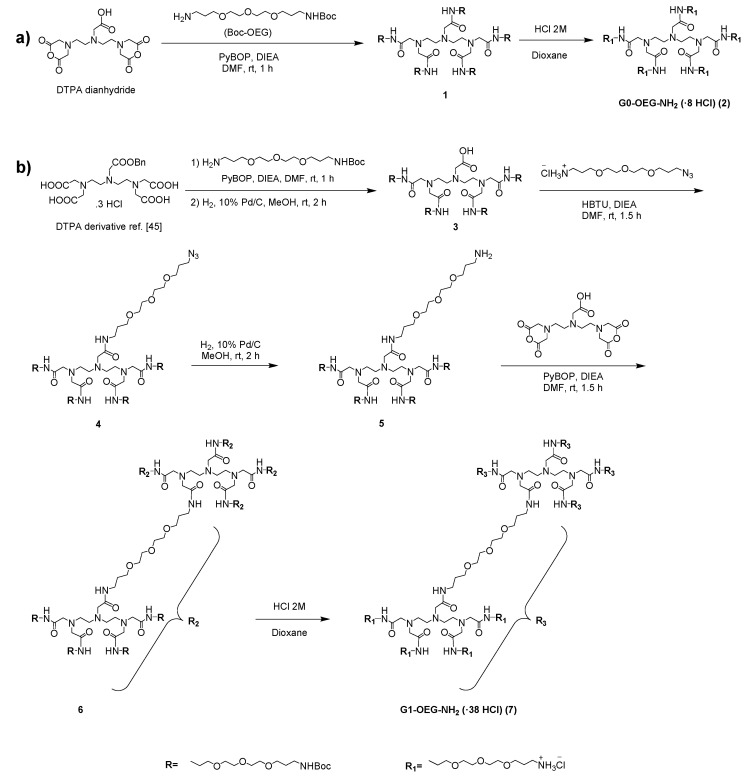
Synthesis of (**a**) G0-OEG-NH_2_ and (**b**) G1-OEG-NH_2_ dendrimers.

G0-OEG-NH_2_ and G1-OEG-NH_2_ dendrimers are based on a diethylenetriaminepentaacetic acid (DTPA) core (Scheme 2). The precursor dendrimer to G0-OEG-NH_2_ with five OEG branches (**1**) was synthesized as previously described [45], and then by subsequent step of Boc removal we obtained the corresponding amine terminated dendrimer (**2**). On the other hand, G1-OEG-NH_2_ (**7**) with 20 OEG branches is a novel dendrimer and its corresponding synthesis, which involved five steps, was much more complex (Scheme 2).

Dendrimer **1** was prepared by acylation of commercially available diethylenetriaminepentaacetic (DTPA) dianhydride with 1-(*tert*-butoxycarbonyl-amino)-4,7,10-trioxa-13-tridecanamine following described procedures [45] (Scheme 2). A subsequent step of Boc removal by treatment with 2 M solution of HCl in dioxane afforded G0-OEG-NH_2_ dendrimer (**2**).

The zero generation dendron **3** was synthesized as previously described [45] and contained four equivalent oligoethylene glycol (OEG) branches, particularly triethylene-glycol-like ones, functionalized on the surface with *tert*-butoxycarbonyl-amino groups and a differentiated position with a free carboxylic acid. The carboxylic acid was derivatized to the amide with a 1-azido-4,7,10-trioxa-13-tridecanamine hydrochloride moiety using 3-[Bis(dimethylamino)methyliumyl]-3H-benzotriazol-1-oxide hexafluorophosphate (HBTU) as activating agent to give **4** with 94% yield. Finally, the azido group was reduced to the corresponding amine by Pd/C-catalyzed hydrogenation, giving compound **5** with 90% yield.

To prepare the first generation (G1) dendrimer **6**, the DTPA dianhydride was used as core and the carboxylic acid moieties were acylated using product **5** and (Benzotriazol-1-yloxy)tripyrrolidinophosphonium hexafluorophosphate (PyBOP) (46% yield). Finally, the removal of Boc groups by treatment with a 2 M solution of HCl in dioxane gave the G1-OEG-NH_2_ dendrimer (**7**) with 99% yield.

### 3.2. Synthesis and Characterization of G0- and G1-OEG-PROXYL Radical Dendrimers

**Scheme 3:**
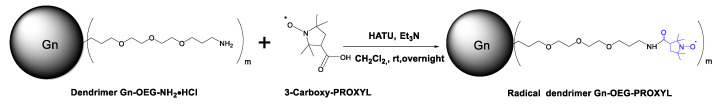
Synthesis of radical dendrimers Gn-OEG-PROXYL, *n* = 0, 1 (m = 5 and m = 20, respectively).

The synthesis of G0- and G1-OEG-PROXYL radical dendrimers were conducted in a similar way (Scheme 3).

The amine groups of G0-/G1-OEG-NH_2_ dendrimers were coupled with the carboxylic acid functional groups of 3-carboxy-PROXYL radicals using HATU as coupling reagent and triethylamine as base, in anhydrous CH_2_Cl_2_ to obtain the corresponding radical dendrimers Gn-OEG-PROXYL, *n* = 0, 1 with amide linkers. Triethylamine was used in excess with both, for the correct activation of the free radical carboxylic acid group by HATU, and to neutralize the hydrochloric acid terminated-NH_2_·HCl end groups of Gn-OEG dendrimers. The purification of both radical dendrimers was performed by ultrafiltration (see Supporting Materials).

Due to the presence of paramagnetic PROXYL units, the full functionalization of radical dendrimers was verified by EPR (see Section 3.3). The purity of the radical dendrimers was verified by SEC-GPC, using water (with 0.25 mM LiCl) as eluent (Figure S1). G1-OEG-PROXYL with a bigger hydrodynamic volume eluted at lower retention time (11.91 min.) than G0-OEG-PROXYL (12.39 min.). By FT-IR (Figure S2) we observed the carbonyl stretching vibrational band from the amide group at 1650 cm^−1^ as well as the corresponding N-H stretching band at 3303 cm^−1^. The bands from the PROXYL radical can be found at 1364 cm^−1^ and 1290 cm^−1^, assigned to the N-O^•^ stretching [47] and -CH- bending, respectively. The band of the ether from the dendrimer branches can be found at 1100 cm^−1^. DLS particle size distribution by volume of Gn-OEG-PROXYL, *n* = 0, 1 radical dendrimers determined in the same conditions revealed a single distribution in each sample, with a mean hydrodynamic diameter of 1.39 ± 0.15 and 4.07 ± 0.33 nm, respectively (see in Figure S3 the particle size distribution by volume, intensity and number of both compounds). By MALDI-TOF it was possible to analyze radical dendrimers of zero generation G0-OEG-PROXYL using dithranol as the matrix, obtaining the corresponding molecular ion peak (see Figure S4). However, it was not possible to analyze with this technique the dendrimer of the first generation G1-OEG-PROXYL with 20 radicals in its periphery, as is common for these types of compounds [48,49]. In addition, the G0-OEG-PROXYL radical dendrimer was treated with ascorbic acid to reduce the PROXYL radical units to their corresponding hydroxylamines (Scheme S1), to eliminate their radical character and characterize them by ^1^H NMR. The ^1^H NMR spectrum lines of G0-OEG-PROXYL-H in CDCl_3_ appeared quite broad, indicating that the radical character was not completely eliminated. However, even in this situation, we could distinguish the different groups of protons of the structure (see Figure S5). A new group of proton signals that did not exist in the initial dendrimer spectrum clearly appeared in the G0-OEG-PROXYL-H spectrum between 0.8 ppm and 1.5 ppm, which corresponded to the protons of PROXYL units. Moreover, the relative integrals of the ^1^H resonances of PROXYL protons and the other groups of protons were consistent with the corresponding theoretical number of protons of the structure (see Figure S6).

### 3.3. EPR Study of G0-OEG-PROXYL and G1-OEG-PROXYL

**Figure 1:**
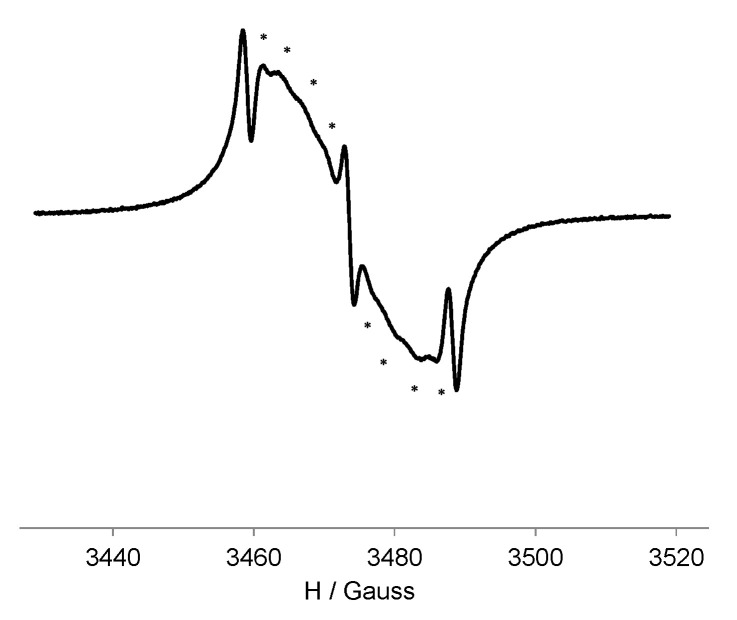
EPR (electron paramagnetic resonance) spectrum of G0-OEG-PROXYL at 300 K in CH_2_Cl_2_ with the eight broad alternate lines marked with asterisks.

The EPR spectrum of the PROXYL free radical presents the typical nitroxide three-line spectrum pattern from the coupling of the unpaired electron with the ^14^N atom of the N-O^•^ unit, with hyperfine coupling constant *a*_N_ ca. 15.6 G. However, the EPR spectrum of G0-OEG-PROXYL at 300 K showed 11 lines separated by ca. 3.0 G with alternating linewidth effect: three narrow lines (1st, 6th and 11th) and in between them two groups of four broad lines (Figure 1). This spectral pattern fits with a spectrum generated by strong spin exchange interaction (|*J*| >> |*a*_N_|) between five nitroxide units (because in the case of two, three or four interacting nitroxide units it would give rise to 5-, 7- and 9-line hyperfine pattern, respectively). In fact, in the case of a flexible polyradical containing five nitroxide radicals with only a through-space spin exchange mechanism between them, we may have two limit cases. When radicals are too far and therefore not interacting among them (|*J*| << |*a*_N_|), the spectrum would be similar to that of five independent monoradicals exhibiting three lines separated by *a*_N_, whereas when radicals are closer and thereby |*J*| >> |*a*_N_|, it would give rise to an eleven-line hyperfine pattern with a separation of 1/5 *a*_N_, as it is our case. However, the relative EPR line intensities observed do not correspond to the theoretical ones expected for five equivalent nitroxyl radicals, since there exists spin exchange dynamics modulated by the temperature. In fact, *J* may not be constant in solution because the radical units could be in movement and consequently, the EPR spectra should be dependent on the conformation and mobility of the dendrimer branches (i.e., on the frequency of collisions of the spin-bearing groups) that depends on the temperature (Figure S7) and solvent nature. At high temperature, the frequency of collisions is higher but as the temperature is gradually lowered (Figure S7), the alternate lines broaden [50] and disappear (leading to three main lines) since the frequency of collisions decreases and the spectra are less affected by spin exchange interaction. Observing 11 lines in the EPR spectrum (and the corresponding spin exchange modulation by temperature) is evidence of the anchoring of nitroxides to the dendrimer branches and the full functionalization of them with five nitroxide units.

**Figure 2:**
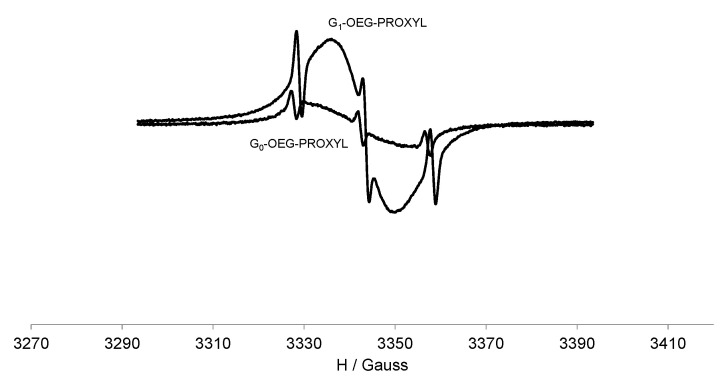
EPR spectra of G0-OEG-PROXYL and G1-OEG-PROXYL at 300 K in CH_2_Cl_2_, at the same concentration 10^−4^ M and in quantitative conditions.

On the other hand, the EPR spectral pattern of G1-OEG-PROXYL at 300 K (Figure 2) is dominated by a non-resolved, single, intense broad line overlapping three narrow lines. This broad line results from spin exchange and dipole–dipole interactions between several nitroxide units anchored on the dendrimer surface, averaged over different interaction distances. In fact, only for compound G0-OEG-PROXYL the spectral resolution allows us to determine the number of interacting PROXYL radicals. However, with 20 radical units the spectral resolution does not allow the observation of the ever decreasing spacing between the various hyperfine transitions (decrease of the splitting value *a_N_*/*n* and increase of the number of lines 2n + 1). We also observed alternating linewidth effect upon cooling from 300 to 220 K (Figure S8) because of the modulation of the spin exchange interaction with the temperature. As the temperature goes down, the single broad line intensity starts to decrease while the three main lines are maintained, as in the zero generation. In addition, the EPR intensity of G1-OEG-PROXYL (measured in terms of area, i.e., double integral of the EPR signal) was exactly 4 times higher than that of G0-OEG-PROXYL in quantitative conditions, confirming the ratio of 20 to 5 PROXYL radical units between both radical dendrimers, respectively (Table 1, Figure 2).

**Figure 3:**
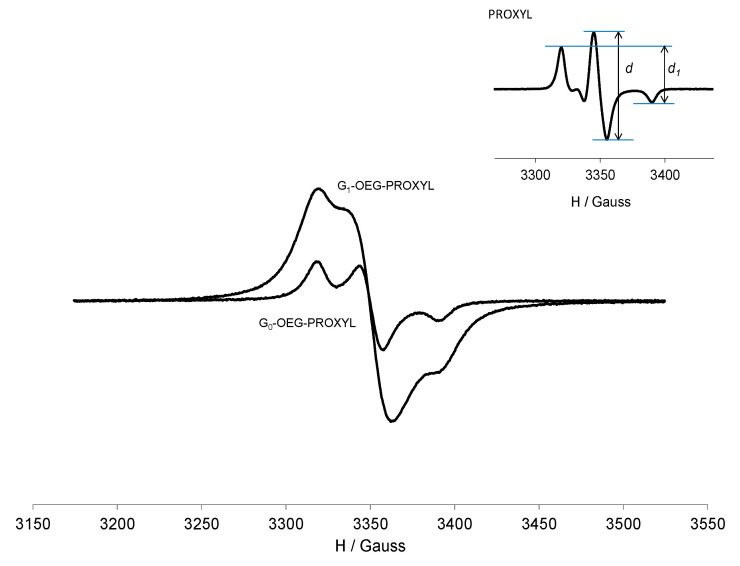
EPR spectra of G0-OEG-PROXYL and G1-OEG-PROXYL at 120 K in CH_2_Cl_2_, at the same concentration 10^−4^ M and in quantitative conditions. Inset: free PROXYL at 120 K with *d* and *d*_1_ representation.

In frozen solution (120 K), the shape of the spectra changes completely as we are under anisotropic conditions (Figure 3). In such conditions, the EPR spectrum is sensitive to the dipole–dipole interaction between neighboring nitroxides which substantially alters the shape of the spectrum. This effect could be estimated by the empirical ratio of peak heights *d*_1_/*d* [51,52]. This parameter is shown to be sensitive to the distance between adjacent nitroxides and hence is a convenient measure of the strength of the dipole–dipole interactions (e.g., the higher the ratio, the shorter the distance between the radical centers and hence the higher the radical interactions). The *d*_1_/*d* parameter for G0 and G1-OEG-PROXYL was 0.71 and 0.87, respectively, whereas for free nitroxide it was only 0.53 [40]. Thus, both radical dendrimers showed significant contribution of intramolecular dipole–dipole interactions among adjacent spin labels, but, as expected, G1 shows much higher *d*_1_/*d* ratio than G0, since the presence of four times more radicals should make them be closer. Furthermore, in frozen solution we observed that the intensity of G1 (double integral) was 4 times higher than that in G0, measured in quantitative conditions (Table 1 and Figure 3).

**Figure 4:**
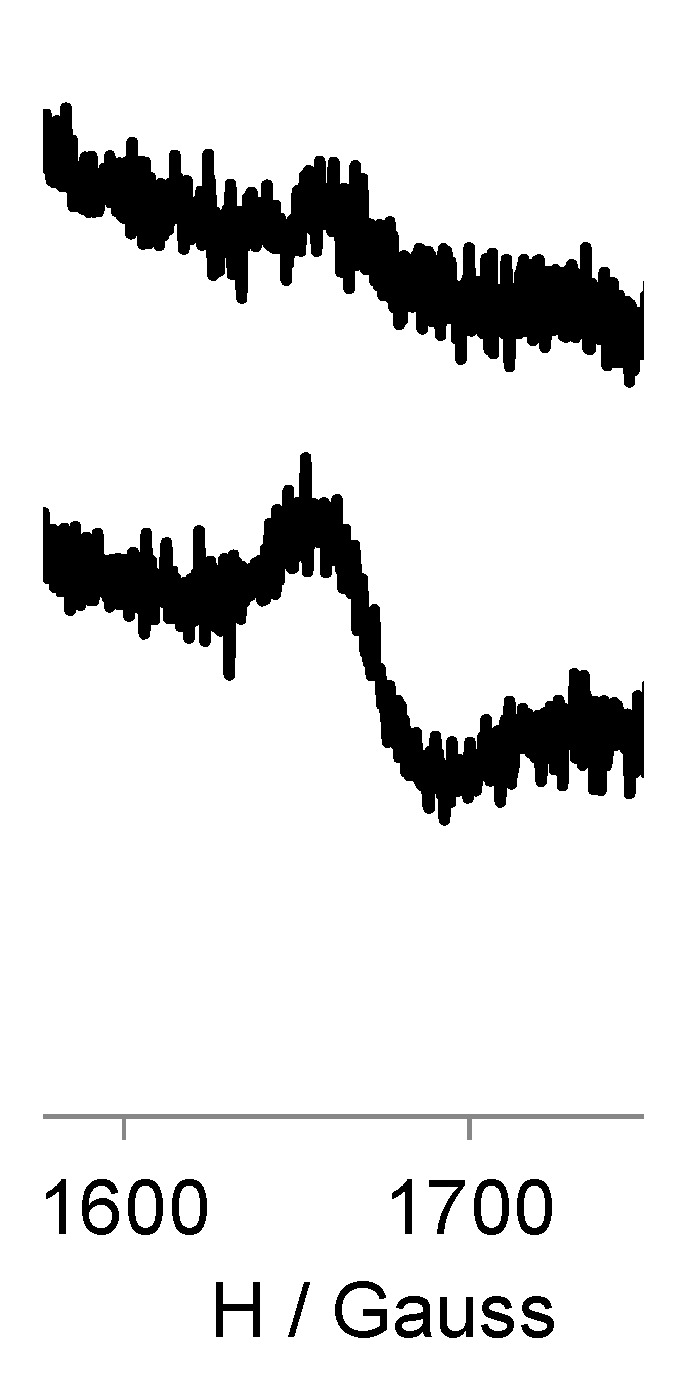
EPR spectra of |Δ*m*_s_| = 2 transition at half-field for the same concentration 10^−4^ M of G0-OEG-PROXYL and G1-OEG-PROXYL at 120 K in CH_2_Cl_2_.

In addition, under frozen conditions we also observed an |Δ*m*_s_| = 2 transition at half-field in both generations, in dilution conditions to ensure the study of the intramolecular interactions present in the dendrimers (Figure 4). This forbidden transition is characteristic of dipolar coupled spins and gives direct evidence of the presence of a high-spin state. No |Δ*m*_s_| = 3 or other lower-field transitions could be observed. We also observed a direct proportionality between the corresponding EPR signal intensity and the number of radicals of each generation.

The EPR spectra of Gn-OEG-PROXYL (*n* = 0, 1) radical dendrimers in water showed similar spectral patterns to organic solvents, although with a higher alternating linewidth effect due to the increase in the solvent polarity, a known effect that lowers the spin exchange interactions [53].

### 3.4. Relaxivity Measurements and Cytotoxicity

Imaging experiments of 3-carboxy-PROXYL and Gn-OEG-PROXYL *n* = 0, 1 radical dendrimers were performed at 7T and room temperature in 30 mM phosphate buffer pH 7.4. In Table 2 *r*_1_ and *r*_2_ relaxivity constants per molecule and per unit of nitroxyl radical, calculated from relaxation times measurements from the imaging experiments, are presented. The plots of proton relaxation rates ^1^H *R*_1,2_ (*R*_1,2_ = 1/*T*_1,2_) of water molecules versus the nitroxyl unit concentrations were linear for all agents, over the concentration range studied (see Tables S1–S3 and Figures S9–S11).

The presence of a high number of paramagnetic nitroxides in the dendrimer periphery resulted in high molecular relaxivities. The *r*_1_ values were scaled with the number of radicals, for this reason the relaxivity per unit of nitroxyl radical was maintained constant from the free PROXYL. The molecular *r*_1_ relaxivity obtained for G0-OEG-PROXYL (Table 2) was 0.91 mM^−1^s^−1^ while for G1-OEG-PROXYL it was 3.4 mM^−1^s^−1^.

It is worth highlighting that G1-OEG-PROXYL presents practically the same relaxivity than the most widely used Gd contrast agent in clinics, Magnevist (Gd-DTPA, 3.2 mM^−1^s^−1^), in the same conditions (r.t., 7T). Moreover, we have achieved this optimal relaxivity with an entirely organic metal-free molecule. The absence of Gd in the radical dendrimer eliminates the risks associated with its unwanted accumulation in the body and the derived health problems. Furthermore, the control over the size of the dendrimers opens the opportunity to modulate their distribution profile in the body, which is impossible in the case of Gd-DTPA. Another important thing to highlight is the biocompatible essence of OEG dendrimers [46], in contrast to Gd-based contrast agents, which make them very interesting for many applications. In addition, nitroxides have been shown minimal in vivo toxicity [54,55]. To really assess the toxicity of G0- and G1-OEG-PROXYL dendrimers, in vitro cell viability assays were conducted with African green monkey kidney (Vero) cell line. In these assays, the cells were incubated with G0- and G1-OEG-PROXYL radical dendrimer at different concentrations ranging from 0.0625 to 2 mM per radical unit for 24 h, by XTT assay. The XTT assay is used to measure cellular metabolic activity as an indicator of cell viability, proliferation and cytotoxicity. This colorimetric assay is based on the reduction of a yellow tetrazolium salt (XTT) to an orange formazan dye by metabolically active cells. As a result, neither the G0- nor the G1-OEG-PROXYL dendrimers showed any cytotoxicity in vitro, in the tested concentration range (Figure S12).

As previously mentioned, there are very few reported works based on dendrimers fully functionalized with nitroxides for MRI applications. However, authors have described water-phase aggregation problems in such nitroxide-based dendrimers, especially in large generation dendrimers. By our strategy of using dendrimers with water-soluble branches themselves we have overcome such water solubility issues and at the same time we have been able to fully functionalize all dendrimer branches with nitroxides.

In general, the optimal characteristics in a contrast agent are to be highly soluble in physiological pH, to present high ^1^H water relaxivities, to not be toxic and to not accumulate in the body. Our radical dendrimers would fulfill such conditions as they are fully water soluble, present the same relaxivity than the golden standard used in clinics and they are entirely organic and biocompatible non-cytotoxic molecules. All this makes these radical dendrimers excellent candidates to be used as contrast agents for MRI. They are shown as potential candidates to be studied as alternatives to Gd-based contrast agents widely used in many current MRI applications such as diagnosis and follow-up of infectious diseases.

## 4. Conclusions

Two generations of biocompatible OEG dendrimers fully functionalized with 5 and 20 PROXYL organic radicals, respectively, were synthesized and characterized with the aim to obtain entirely organic metal-free paramagnetic species to replace gadolinium-based contrast agents, which have shown residual toxic Gd ion accumulation in the body. Highly water-soluble radical dendrimers were obtained by synthesizing dendrimers with highly water-soluble branches themselves, formed by oligoethylene glycol structures, and they were shown to not be cytotoxic. Importantly, the first G1-OEG-PROXYL radical dendrimer generation had a similar *r*_1_ relaxivity value (3.4 mM^−1^s^−1^) in comparison to Gd-DTPA used in clinics (3.2 mM^−1^s^−1^, r.t. 7T) but in this case avoiding the toxicity risks associated with the unwanted accumulation of Gd in the body. These radical dendrimers are shown as potential candidates to be studied as alternatives to the Gd-based contrast agents widely used in many current MRI applications such as diagnosis and follow-up of infectious diseases.

## Figures and Tables

**Table 1:** Ratio between the relative double integral values of G1 and G0 from their corresponding spectra obtained at 300 and 120 K in quantitative conditions.

Dendrimer	Area (Double Integral) Spectra at 300 K ^a^	Ratio G1/G0	Area (Double Integral) Spectra at 120 K ^a^	Ratio G1/G0
G0-OEG-PROXYL	1.829 × 10^5^	3.96	1.231 × 10^6^	4.08
G1-OEG-PROXYL	7.246 × 10^5^		5.028 × 10^6^	

^a^ The acquisition EPR parameters are the same at each temperature but not between the different temperatures.

**Table 2:** Longitudinal *r*_1_ relaxivity constants per molecule and per unit of nitroxyl radical of G0- and G1-OEG-PROXYL radical dendrimers compared with 3-carboxy-PROXYL, determined at 7T in 30 mM phosphate buffer pH 7.4, 300 K.

Compound	*r*_1_^7T^ (mM^−1^s^−1^) per Molecule	*r*_1_^7T^ (mM^−1^s^−1^) per Unit of Radical	*r*_2_^7T^ (mM^−1^s^−1^) per Molecule	*r*_2_^7T^ (mM^−1^s^−1^) per Unit of Radical
3-carboxy PROXYL	0.18	0.18	0.20	0.20
G0-OEG-PROXYL	0.91	0.18	0.95	0.19
G1-OEG-PROXYL	3.39	0.17	4.02	0.19

## Supplementary Materials

The following are available online at https://www.mdpi.com/1999-4923/12/8/772/s1, Synthesis of G0/G1-OEG-NH_2_·(HCl) and G0/G1-OEG-PROXYL, Figure S1: SEC-GPC of G0-OEG-PROXYL and G1-OEG-PROXYL, Figure S2: FT-IR (ATR) spectra of G0-OEG-PROXYL and G1-OEG-PROXYL, Figure S3: DLS of G0-OEG-PROXYL and G1-OEG-PROXYL, Figure S4: MALDI-TOF of G0-OEG-PROXYL, Figure S5: ^1^H NMR spectra of G0-OEG-NH_2_·(HCl) and G0-OEG-PROXYL-H dendrimers, Figure S6: ^1^H NMR spectrum of G0-OEG-PROXYL-H with the relative integral values, Figure S7: Variable temperature EPR spectra of G0-OEG-PROXYL, Figure S8: Variable temperature EPR spectra of G1-OEG-PROXYL, Table S1: MRI data of PROXYL in PBS, Figure S9: Plots of R_1_ of water molecules versus PROXYL concentration, Table S2: MRI data of G0-OEG-PROXYL in PBS, Figure S10: Plots of R_1_ of water molecules versus the nitroxyl radical unit (PROXYL) concentration and the G0-OEG-PROXYL molecular concentration, Table S3: MRI data of G1-OEG-PROXYL in PBS, Figure S11: Plots of R_1_ of water molecules versus the nitroxyl radical unit (PROXYL) concentration and the G1-OEG-PROXYL molecular concentration. Figure S12: In vitro cytotoxicity assays of G0-OEG-PROXYL and G1-OEG-PROXYL dendrimers.
