# Supplementary material for: Radical Dendrimers Based on Biocompatible Oligoethylene Glycol Dendrimers as Contrast Agents for MRI
Source: Pharmaceutics. 2020 Aug 14;12(8):772. doi: 10.3390/pharmaceutics12080772 (PMC7464757; doi:10.3390/pharmaceutics12080772)
Supplement: Supplementary file 1 [file pharmaceutics-12-00772-s001.pdf]

pharmaceutics

Supplementary Materials: Radical Dendrimers Based on Biocompatible Oligoethylene Glycol Dendrimers as Contrast Agents for MRI

Songbai Zhang, Vega Lloveras, Daniel Pulido, Flonja Liko, Luiz F. Pinto Fernando Albericio, Miriam Royo and José Vidal-Gancedo *

Synthesis of G0-OEG-NH_2 and G1-OEG-NH_2 Dendrimers

Synthesis of Compound 1

Dendrimer 1 was prepared by acylation of commercially available diethylenetriaminepentaacetic (DTPA) dianhydride with 1-(*tert*-butoxycarbonyl-amino)-4,7,10-trioxa-13-tridecanamine following described procedures [1].

Synthesis of G0-OEG-NH_2(. 8 HCl) Dendrimer (2)

Compound 1 (126 mg, 66.2 µmol) was dissolved in dioxane (2 mL) and a 4 M solution of HCl in dioxane (2 mL) was added. The resulting mixture was stirred at room temperature for 1 h. Then, the mixture was evaporated to dryness. Finally, the crude was dissolved in water (5 mL) and lyophilized to obtain compound 2 (111 mg, 65.6 µmol, 99%). ^{1}H NMR (400 MHz, D_2O) δ 4.02 (s, 8H), 3.72 – 3.61 (m, 52H), 3.56 (t, *J* = 6.4 Hz, 10H), 3.48 (t, *J* = 6.4 Hz, 4H), 3.36 – 3.19 (m, 14H), 3.10 (t, *J* = 7.2 Hz, 10H), 2.00 – 1.90 (m, 10H), 1.86 – 1.75 (m, 10H). ^{13}C NMR (101 MHz, D_2O) δ 169.5, 166.5, 69.5, 69.4, 69.3, 69.2, 68.2, 56.5, 55.0, 52.6, 50.3, 37.6, 36.6, 28.1, 26.4. LRMS: calculated mass for $C_{64}H_{141}Cl_8N_{13}O_{20}$ (8 HCl) 1691.8, calculated mass for $C_{64}H_{134}N_{13}O_{20}$ (amine) 1405.0 $[M+H]^+$, found by HPLC-MS (ESI) 1404.8, 703.3 $[M + 2H]^{2+}$, 469.1 $[M + 3H]^{3+}$, 352.1 $[M + 4H]^{4+}$.

Synthesis of Compound 3 and 4

Compound 3 was synthesized as described in [1], starting from 4-benzyloxycarbonylmethyl-1,1,7,7-tetra(carboxymethyl)-1,4,7-triazaheptane, a DTPA synthetic derivative. Compound 3 (444 mg, 277 µmol, 1.0 equiv) was dissolved in DMF (5 mL) and *N*,*N*-Diisopropylethylamine (DIEA) (0.19 mL, 1.12 mmol, 4.0 equiv) and 3-[Bis(dimethylamino)methyliumyl]-3H-benzotriazol-1-oxide hexafluorophosphate (HBTU) (132 mg, 348 µmol, 1.2 equiv) were added. Then, after stirring 2 min at room temperature a solution of 1-azido-4,7,10-trioxa-13-tridecanamine hydrochloride (98.1 mg, 347 µmol, 1.2 equiv) in DMF (2 mL) was added. The resulting mixture was stirred at room temperature for 90 min. After this time the solvent was evaporated to dryness. The crude was dissolved in AcOEt (30 mL) and washed with saturated $NaHCO_3$ (3 × 30 mL), 1 M HCl (3 × 30 mL) and brine (1 × 30 mL). The organic phase was dried over $MgSO_4$ and evaporated to obtain the desired compound 4 (478 mg, 261 µmol, 94%). ^{1}H NMR (400 MHz, $CDCl_3$) δ 7.60 (bs, NH), 7.37 (bs, NH), 5.06 (bs, NH), 3.64 – 3.44 (m, 60H), 3.39 – 3.24 (m, 12H), 3.23 – 3.08 (m, 16H), 3.04 (s, 2H), 2.66 – 2.50 (m, 8H), 1.85 – 1.66 (m, 20H), 1.39 (s, 36H). ^{13}C NMR (101 MHz, $CDCl_3$) δ 171.1, 170.7, 156.1, 78.9, 70.6, 70.5, 70.3, 70.2, 70.2, 69.5, 67.9, 59.2, 53.4, 53.3, 48.5, 38.5, 37.2, 29.8, 29.5, 29.1, 28.5. LRMS: calculated mass for $C_{84}H_{164}N_{15}O_{28}$ 1831.2 $[M+H]^+$, found by HPLC-MS (ESI) 1832.2, 916.5 $[M + 2H]^{2+}$.

Synthesis of Compound 5

Compound 4 (218 mg, 119 µmol) was dissolved in MeOH (15 mL) and 10% Pd/C (22 mg, 10% *w*/*w*) was added. The resulting suspension was stirred at room temperature for 2 h under H_2

atmosphere. The catalyst was removed by filtration through Celite and the solvent was evaporated to dryness to afford compound 5 (193 mg, 107 μmol, 90%). ^{1}H NMR (400 MHz, $CDCl_3$) δ 7.77 – 7.58 (m, NH), 5.07 (bs, NH), 3.633.52 (m, 42H), 3.52 – 3.43 (m, 20H), 3.35 – 3.24 (m, 10H), 3.22 – 3.10 (m, 16H), 3.05 (s, 2H), 2.89 (t, *J* = 6.3 Hz, 2H), 2.67 – 2.51 (m, 8H), 1.82 – 1.67 (m, 20H), 1.40 (s, 36H). ^{13}C NMR (101 MHz, $CDCl_3$) δ 171.6, 170.9, 156.2, 78.9, 70.6, 70.5, 70.2, 70.2, 70.0, 70.0, 69.8, 69.5, 69.4, 69.1, 59.2, 58.6, 53.4, 53.3, 40.0, 38.5, 37.1, 36.7, 29.8, 29.6, 29.5, 28.5. LRMS: calculated mass for $C_{84}H_{166}N_{13}O_{28}$ 1805.2 $[M+H]^+$, found by HPLC-MS (ESI) 1805.2, 903.5 $[M + 2H]^{2+}$, 602.8 $[M + 3H]^{3+}$.

Synthesis of Compound 6

To a solution of diethylenetriaminepentaacetic dianhydride (5.7 mg, 15.9 μmol, 1 equiv) and 5 (193 mg, 107 μmol, 6.7 equiv) in DMF (5 mL) were added (Benzotriazol-1-yloxy)tripyrrolidinophosphonium hexafluorophosphate (PyBOP) (37.5 mg, 72.0 μmol, 4.5 equiv) and DIEA (24 μL, 141 μmol, 8.9 equiv). The resulting mixture was stirred at room temperature for 90 min. After this time the crude was evaporated to dryness. The crude was dissolved in CH_2Cl_2 (25 mL) and washed with saturated $NaHCO_3$ (2 × 25 mL) and brine (1 × 25 mL). The organic phase was dried over $MgSO_4$ and evaporated. The resulting crude was purified by semipreparative reversed-phase HPLC (70–78% acetonitrile in aqueous 10 mM NH_4HCO_3 in 8 min, XBridge C_{18} 19 mm × 150 mm 5 μm), affording compound 6 (67.7 mg, 7.26 μmol, 46%). ^{1}H NMR (400 MHz, $CDCl_3$) δ 7.88 (bs, NH), 5.08 (bs, NH), 3.63 – 3.40 (m, 322H), 3.40 – 3.05 (m, 186H), 1.80 – 1.62 (m, 100H), 1.36 (s, 180H). ^{13}C NMR (101 MHz, $CDCl_3$) δ 170.8, 156.2, 79.0, 70.6, 70.6, 70.3, 70.2, 69.5, 69.3, 58.7, 53.4, 38.5, 37.2, 29.8, 29.5, 28.6. LRMS: calculated mass for $C_{434}H_{839}N_{68}O_{145}$ 9325.0 $[M+H]^+$, found by HPLC-MS (ESI) 1866.7 $[M + 5H]^{5+}$, 1555.8 $[M + 6H]^{6+}$, 1333.7 $[M + 7H]^{7+}$, 1167.1 $[M + 8H]^{8+}$, 1037.6 $[M + 9H]^{9+}$.

Synthesis of G1-OEG-NH_2(. 38 HCl) Dendrimer (7)

Compound 6 (67.7 mg, 7.26 μmol) was dissolved in dioxane (2 mL) and a 4 M solution of HCl in dioxane (2 mL) was added. The resulting mixture was stirred at room temperature for 2 h. Then, the mixture was evaporated to dryness. Finally, the crude was dissolved in water (5 mL) and lyophilized to obtain compound 7 (63.2 mg, 7.25 μmol, >99%). ^{1}H NMR (400 MHz, D_2O) δ 3.94 – 3.83 (m, 40H), 3.75 – 3.63 (m, 260H), 3.58 (t, *J* = 6.4 Hz, 68H), 3.45 – 3.37 (m, 20H), 3.37 – 3.24 (m, 80H), 3.13 (t, *J* = 7.2 Hz, 40H), 2.02 – 1.92 (m, 40H), 1.89 – 1.76 (m, 60H). ^{13}C NMR (101 MHz, D_2O) δ 168.1, 69.5, 69.5, 69.3, 69.3, 68.2, 56.9, 52.1, 51.2, 37.6, 36.5, 28.2, 26.4. LRMS: calculated mass for $C_{334}H_{716}Cl_{38}N_{68}O_{105}$ 8690.1 (38 HCl), calculated mass for $C_{334}H_{679}N_{68}O_{105}$ (amine) 7324.0 $[M+H]^+$, found by HPLC-MS (ESI) 1047.7 $[M + 7H]^{7+}$, 916.9 $[M + 8H]^{8+}$, 815.1 $[M + 9H]^{9+}$, 733.7 $[M + 10H]^{10+}$, 667.2 $[M + 11H]^{11+}$, 611.6 $[M + 12H]^{12+}$, 564.7 $[M + 13H]^{13+}$, 524.5 $[M + 14H]^{14+}$, 489.6 $[M + 15H]^{15+}$.

Synthesis of G0-OEG-PROXYL Radical Dendrimers

3-carboxy-proxyl (25.312 mg; 1.3 eq. per group) and HATU (52.19 mg; 1.3 eq. per group) were dissolved in anhydrous DCM (2 mL) in a 25 mL round flask. Then, triethylamine (71 μL; 25 eq.) was added with a syringe and it was let to stir at room temperature for 15 minutes. In another 25 mL round flask under an argon atmosphere, G0-OEG-NH_2(.8 HCl) (2) dendrimer (50.0 mg; 1 eq.) was dissolved in anhydrous DCM (2 mL). Then, triethylamine (80 μL; 28 eq.) was added with a syringe and was let to stir for 15 minutes. Subsequently, the mixture of the first flask containing 3-carboxy-proxyl was transferred to the second one containing the G0-OEG-NH_2 dendrimer. The reaction mixture was let to stir at room temperature overnight. The reaction was monitored by TLC using ninhydrin. The product was purified by ultrafiltration in a mixture of water/acetone (10%/90%), and obtained with a 61% yield. The full radical functionalization was verified by EPR and its purity by SEC (see Section 3.2 and 3.3. and the supporting information). For ^{1}H NMR characterization see Figure S3 and S4. MALDI-TOF MS (dithranol, linear mode m/z): calculated mass for $C_{109}H_{203}N_{18}O_{30}$: 2245.92; found: 2247.31 $[M+H]^+$. IR (ATR, cm^{-1}): 3303 (-N-H-)st; 1650 (C=O)st; 1364 (N-O.)st; 1290 (-C-H-)bend; 1100 (-C-O-C-)st.

Synthesis of G1-OEG-PROXYL Radical Dendrimers

3-carboxyl-PROXYL (13.00 mg, 1.5 eq. per group) and HATU (26.1 mg, 1.5 eq. per group) were dissolved in 3 mL of anhydrous DCM in a round flask, then triethylamine (50 μL; 154 eq.) was added and it was let to stir at room temperature for 30 min. G2-OEG-NH_2(. 38 HCl) (**7**) dendrimer (17 mg; 1 eq.) was dissolved in 3 mL of DCM in another round flask. The solution containing 3-carboxyl-PROXYL was transferred into the flask containing G2-OEG-NH_2 dendrimer and the reaction mixture was let to stir at room temperature overnight. The reaction was monitored by TLC using ninhydrin. The product was purified by ultrafiltration in water, and obtained with a 54% yield. The full radical functionalization was verified by EPR and its purity by SEC (see Section 3.2 and 3.3. and the supporting information). IR (ATR, cm^{-1}): 3303 (-N-H-)st; 1650 (C=O)st; 1364 (N-O.)st; 1290 (-C-H-)bend; 1100 (-C-O-C-)st.

SEC-GPC of G0-OEG-PROXYL and G1-OEG-PROXYL

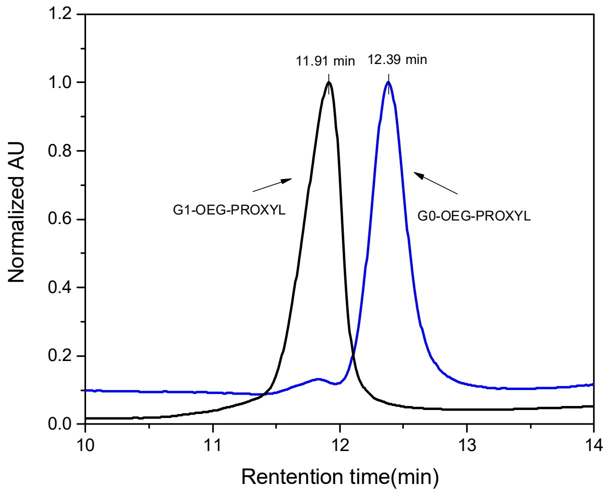

Figure S1. SEC-GPC of G0-OEG-PROXYL and G1-OEG-PROXYL.

FT-IR (ATR) of G0-OEG-PROXYL and G1-OEG-PROXYL

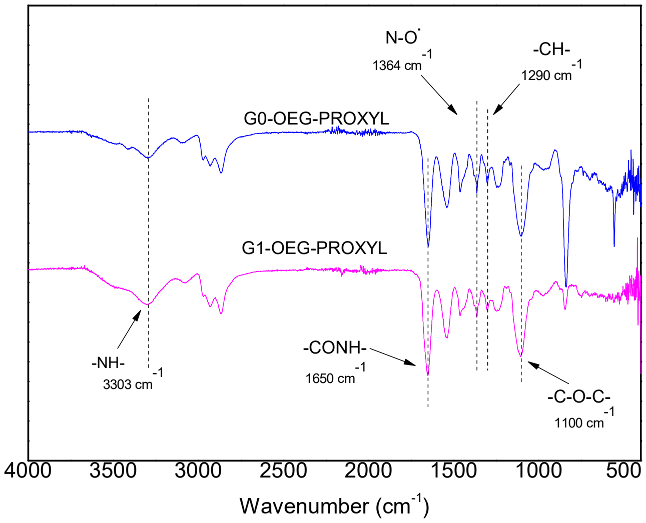

Figure S2. FT-IR (ATR) spectra of G0-OEG-PROXYL and G1-OEG-PROXYL.

DLS of G0-OEG-PROXYL and G1-OEG-PROXYL

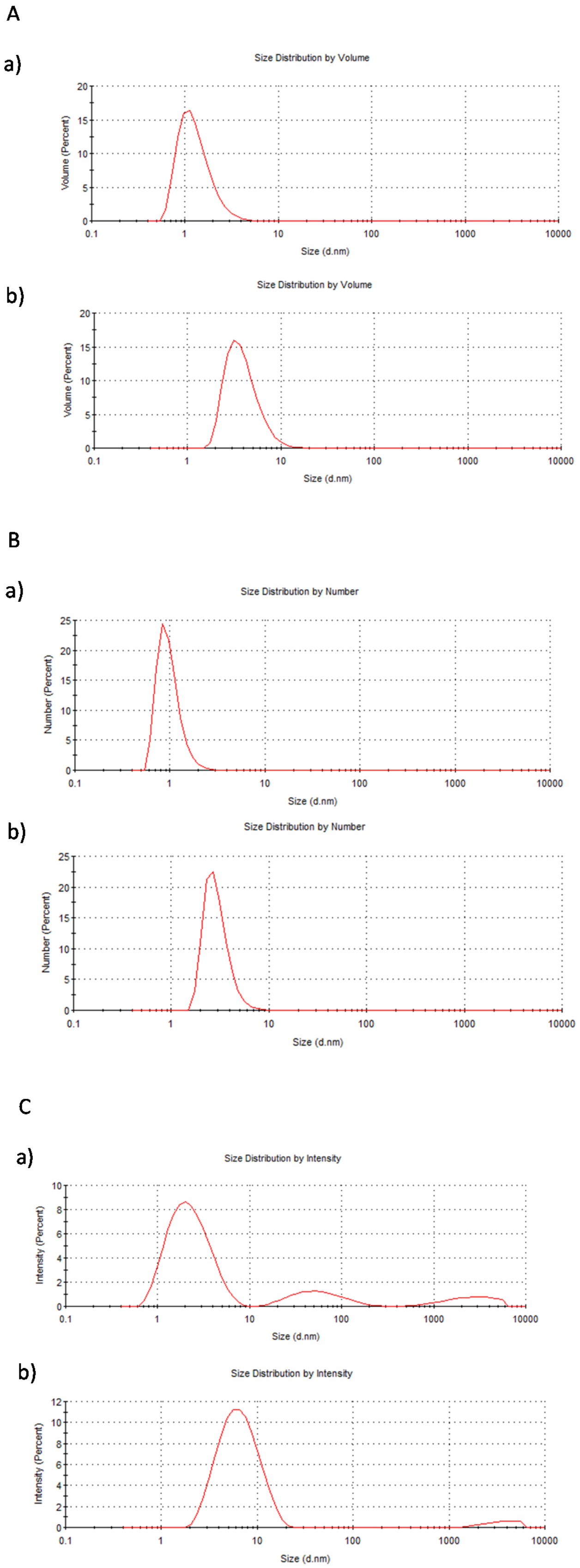

Figure S3. Mean diameters of G0-OEG-PROXYL (**a**) and G1-OEG-PROXYL (**b**) determined by DLS, from the size distribution by volume (**A**), by number (**B**) and by intensity (**C**) at 25 °C in PBS.

MALDI-TOF of G0-OEG-PROXYL

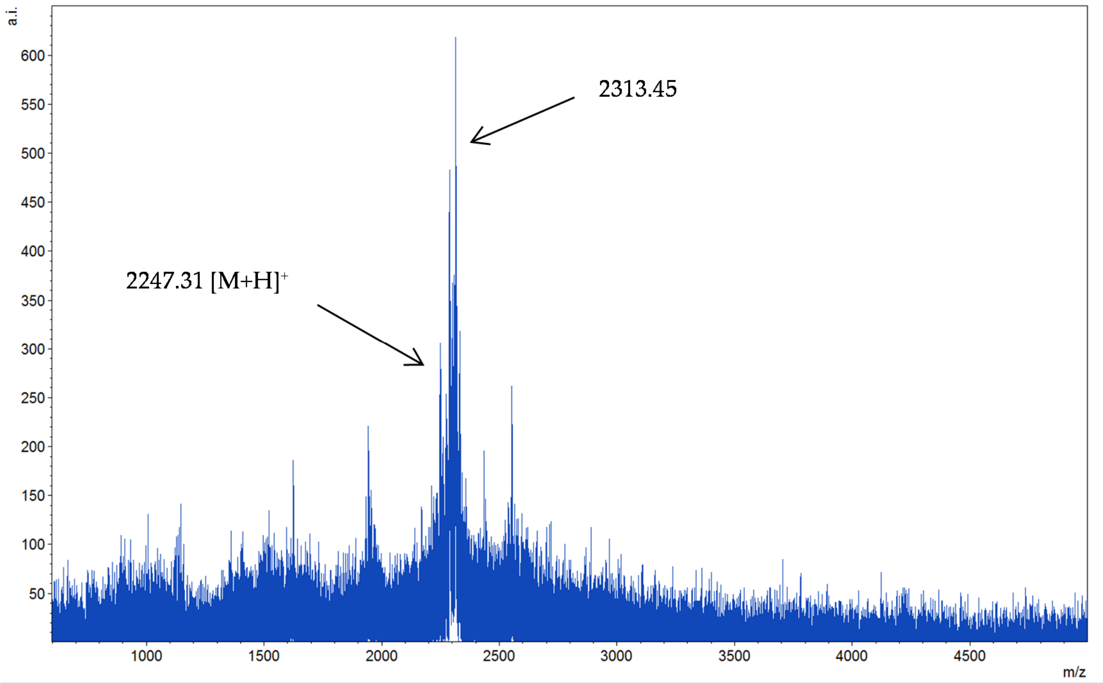

Figure S4. MALDI-TOF of G0-OEG-PROXYL using dithranol matrix.

Reaction of G0-OEG-PROXYL with Ascorbic Acid and ^{1}H NMR of G0-OEG-PROXYL-H

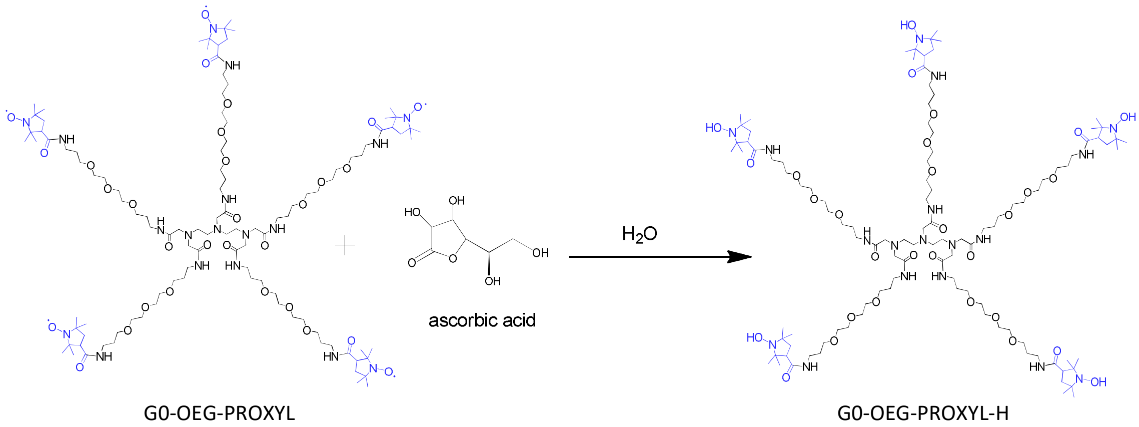

Scheme S1. Reaction of G0-OEG-PROXYL with ascorbic acid.

G0-OEG-PROXYL radical dendrimer (25.86 mg; 1 eq.) was dissolved in H_2O (2mL) in a 10 mL round flask. Subsequently, ascorbic acid excess (103.6 mg; 10 eq. per group) was added and it was let stirring for around 6 h. The product (G0-OEG-PROXYL-H) was purified by ultrafiltration in water/acetone mixture (10%/90%) giving quantitative yield.

In Figure S5b it is shown the corresponding ^{1}H NMR spectrum obtained compared with the initial G0-OEG-NH_2.(8 HCl) dendrimer. It can be observed the appearance of new peaks between 0.8 and 1.5 ppm in the G0-OEG-PROXYL-H spectrum that are absent in the initial G0-OEG-NH_2.(8 HCl), which correspond to the protons of PROXYL units (Figure S5a). In addition, the ratio of the relative integral values between the group of protons (A,B,C + a,b) and the rest of protons (c-m) was the same than the theoretical one: theoretical 18: 22; found 18:21.1, as can be observed in Figure S6.

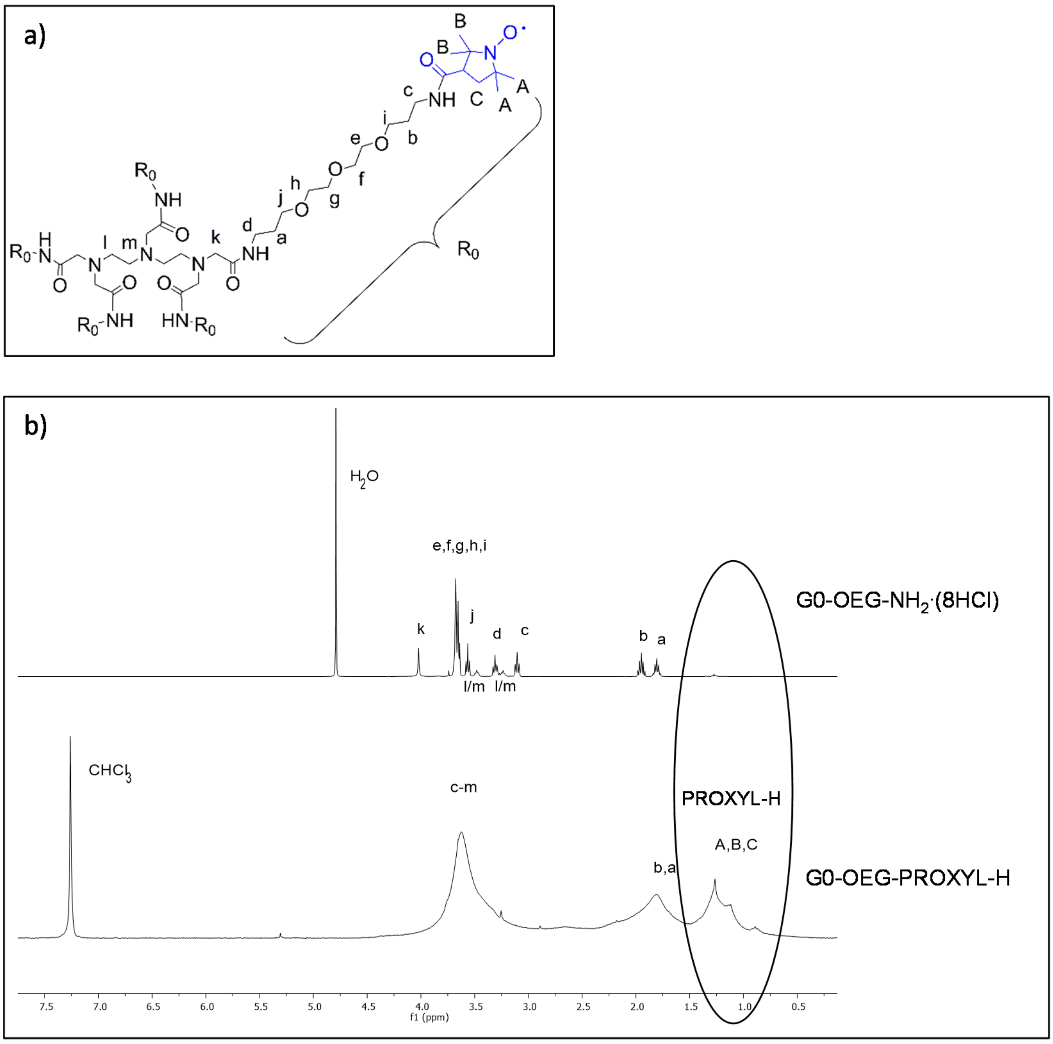

Figure S5. a) Structure of G0-OEG-PROXYL with the protons labelling. **b**) ^{1}H NMR spectra of G0-OEG-NH_2(8 HCl) dendrimer (up) and G0-OEG-PROXYL-H dendrimer (down) with their corresponding peaks assignment.

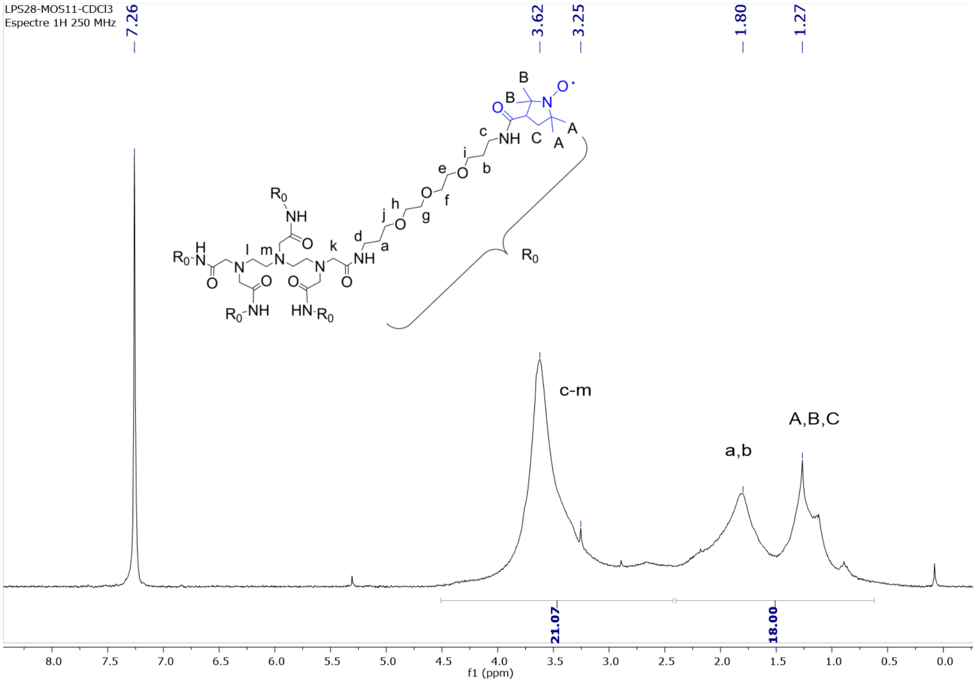

Figure S6. ^1H NMR spectrum of G0-OEG-PROXYL-H dendrimer with their corresponding peaks assignment and relative integral values. The relative integral value for the group of protons (**A,B,C** + **a,b**) was found to be 18 and for the group of protons (c-m) 21.1, the same than the theoretical one 18: 22.

Variable T EPR spectra of G0- and G1-OEG-PROXYL

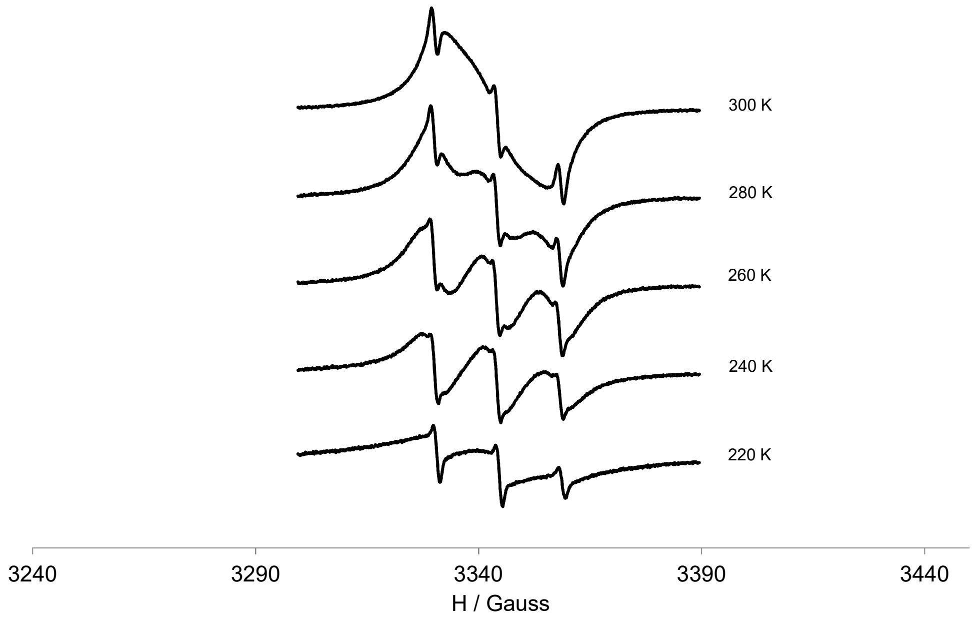

Figure S7. Variable temperature EPR spectra of G0-OEG-PROXYL from 300 to 220 K in CH_2Cl_2.

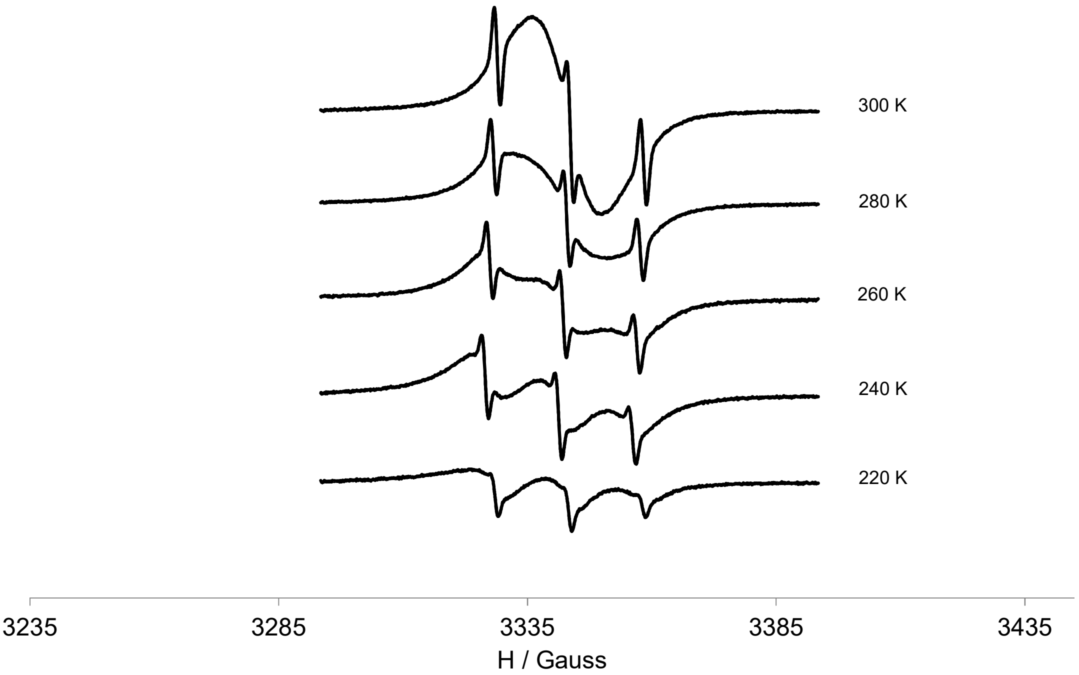

Figure S8. Variable temperature EPR spectra of G1-OEG-PROXYL from 300 to 220 K in CH_2Cl_2.

MRI Data

Table S1. MRI data of PROXYL in PBS.

Conc. radical (mM)	T_1 (ms)	SD (±)	R_1 (s^{-1})
2.83	1049	46	0.953
2.12	1176	44	0.850
1.42	1342	49	0.745
0.71	1651	58	0.606
0.09	2182	103	0.458

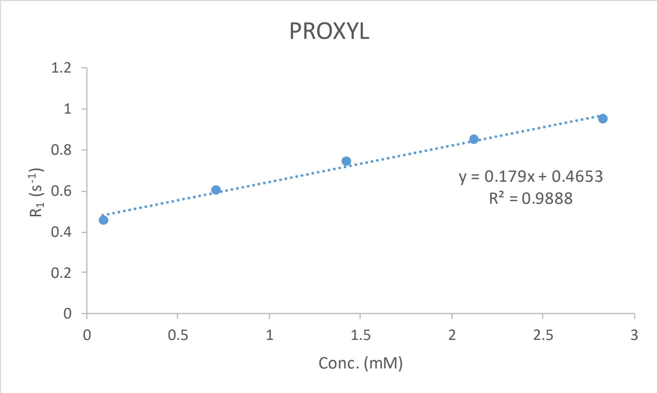

Figure S9. Plots of R_1 of water molecules versus PROXYL concentration.

Table S2. MRI data of G0-OEG-PROXYL in PBS.

Conc. radical (mM)	Conc. molecule (mM)	T_1 (ms)	SD (±)	R_1 (s^{-1})
2.99	0.60	1023	38	0.978
2.24	0.45	1175	48	0.851
1.49	0.30	1489	101	0.672
0.75	0.15	1765	91	0.567
0.37	0.075	1960	85	0.510

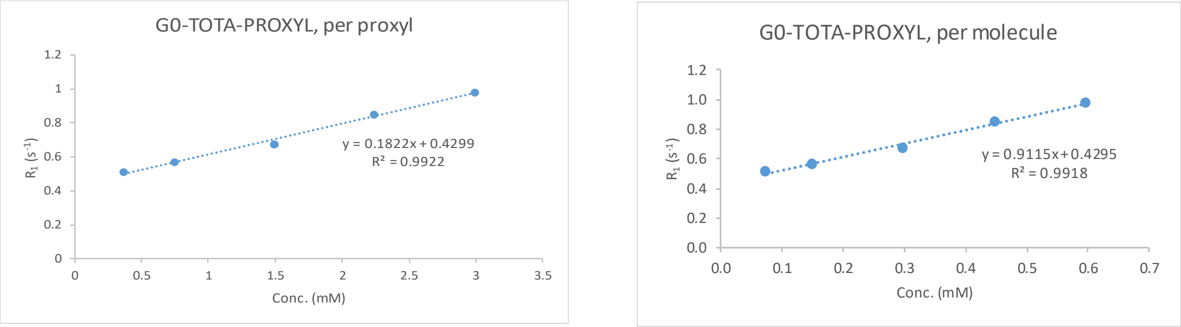

Figure S10. Plots of R_1 of water molecules versus the nitroxyl radical unit (PROXYL) concentration (left) and the G0-OEG-PROXYL molecular concentration (right).

Table S3. MRI data of G1-OEG-PROXYL in PBS.

Conc. molecule (mM)	Conc. radical (mM)	T_1 (ms)	SD (±)	R_1 (s^{-1})
0.303	6.062	690	26	1.449
0.152	3.031	1050	39	0.952
0.114	2.273	1186	46	0.843
0.038	0.758	1935	133	0.517
0.019	0.379	1950	114	0.513

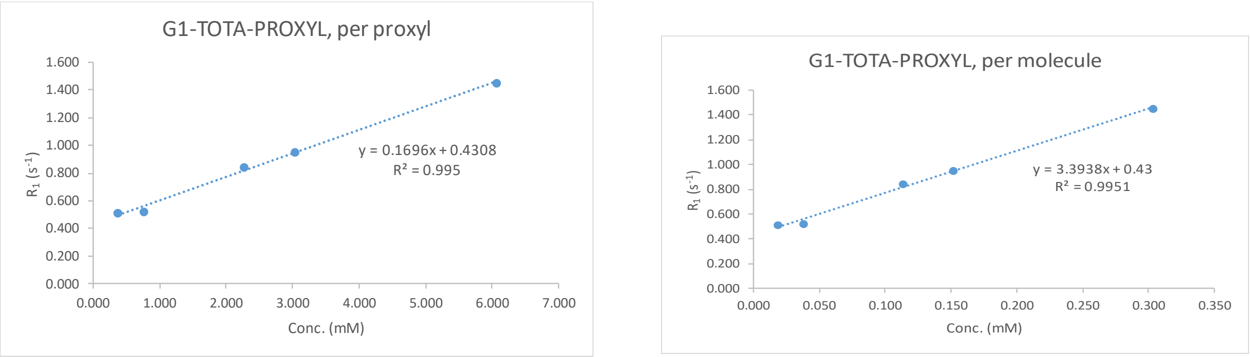

Figure S11. Plots of R1 of water molecules versus the nitroxyl radical unit (PROXYL) concentration (left) and the G1-OEG-PROXYL molecular concentration (right).

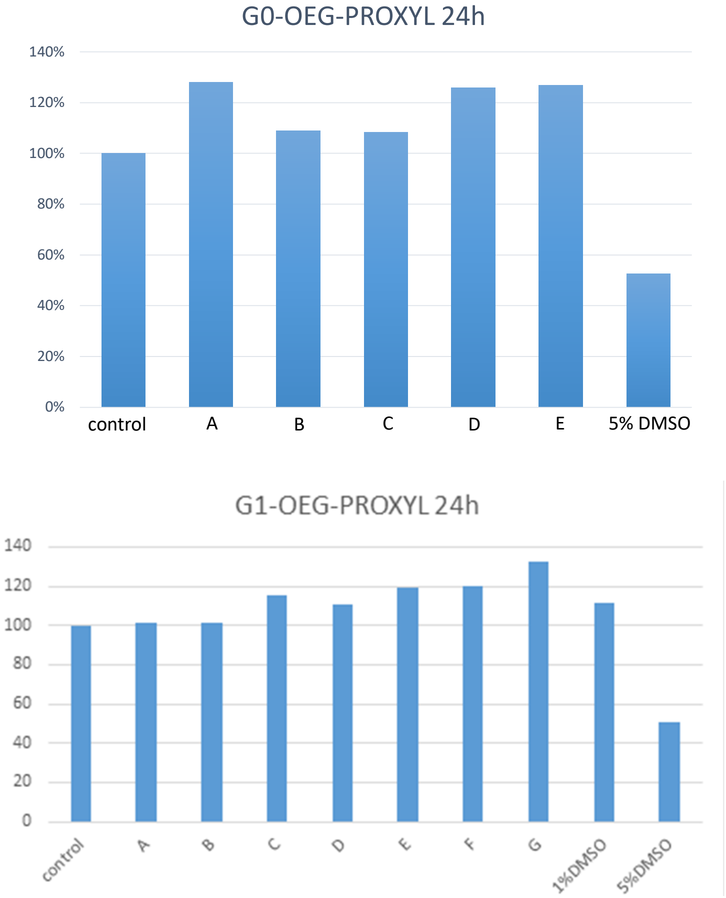

Figure S12. In vitro XTT cell viability assays conducted with African green monkey kidney (Vero) cells incubated with G0-OEG-PROXYL (up) and G1-OEG-PROXYL (down) dendrimers in a concentration of A) and B) 2mM per radical unit, C) 1mM, D) 0.5 mM, E) 0.25 mM, F) 0.125 mM, G) 0.0625 mM, for 24 h.

References

1. Pulido, D.; Albericio, F.; Royo, M. Controlling Multivalency and Multimodality: Up to Pentamodal Dendritic Platforms Based on Diethylenetriaminepentaacetic Acid Cores. *Org. Lett.* **2014**, *16*, 1318–1321.
